# Does movement matter in people with back pain? Investigating ‘atypical’ lumbo-pelvic kinematics in people with and without back pain using wireless movement sensors

**DOI:** 10.1186/s12891-018-2387-x

**Published:** 2019-01-18

**Authors:** Robert A. Laird, Jennifer L. Keating, Kasper Ussing, Paoline Li, Peter Kent

**Affiliations:** 1 Department of Physiotherapy, Monash University, PO Box 527, Frankston, Victoria 3199 Australia; 2 Spine Centre of Southern Denmark, Hospital of Lillebaelt, Middelfart, Denmark; 3 SuperSpine Physiotherapy, 380 Springvale Rd, Forest Hill, Melbourne, 3131 Australia; 4 School of Physiotherapy and Exercise Science, Curtin University, Perth, Australia; 5 Department of Sports Science and Clinical Biomechanics, University of Southern Denmark, Odense, Denmark

**Keywords:** Low back pain, Movement disorders, Range of movement (ROM), Flexion relaxation, Lumbo-pelvic rhythm, Velocity, Assessment

## Abstract

**Background:**

Interventions for low back pain (LBP) commonly target ‘dysfunctional’ or atypical lumbo-pelvic kinematics in the belief that correcting aberrant movement improves patients’ pain and activity outcomes. If atypical kinematic parameters and postures have a relationship to LBP, they could be expected to more prevalent in people with LBP compared to people without LBP (NoLBP). This exploratory study measured, defined and compared atypical kinematic parameters in people with and without LBP.

**Methods:**

Wireless inertial motion and EMG sensors were used to measure lumbo-pelvic kinematics during standing trunk flexion (range of motion (ROM), timing, sequence coordination, and extensor muscle activation) and in sitting (relative sitting position, pelvic tilt range) in a sample of 126 of adults without LBP and 140 chronic LBP subjects. Atypical movement was defined using the 10th/90th centiles of the NoLBP group. Mean differences and prevalence rates for atypical movement were calculated. Dichotomised pain scores for ‘high-pain-on-bending’ and ‘high-pain-on-sitting’ were tested for their association with atypical kinematic variables.

**Results:**

For standing flexion, significant mean differences, after adjusting for age and gender factors, were seen for the LBP group with (i) reduced ROM (trunk flexion (NoLBP 111^o^, LBP 93^o^, *p* < .0001), lumbar flexion (NoLBP 52^o^, LBP 46^o^, *p* < .0001), pelvic flexion (NoLBP 59^o^, LBP 48^o^, *p* < .0001), (ii) greater extensor muscle activation for the LBP group (NoLBP 0.012, LBP 0.25 *p* < .0001), (iii) a greater delay in pelvic motion at the onset of flexion (NoLBP − 0.21 s; LBP − 0.36 s, *p* = 0.023), (iv) and longer movement duration for the LBP group (NoLBP 2.28 s; LBP 3.18 s, *p* < .0001). Atypical movement was significantly more prevalent in the LBP group for small trunk (× 5.4), lumbar (× 3.0) and pelvic ROM (× 3.9), low FRR (× 4.9), delayed pelvic motion at 20^o^ flexion (× 2.9), and longer movement duration (× 4.7). No differences between groups were seen for any sitting parameters. High pain intensity was significantly associated with small lumbar ROM and pelvic ROM.

**Conclusion:**

Significant movement differences during flexion were seen in people with LBP, with a higher prevalence of small ROM, slower movement, delayed pelvic movement and greater lumbar extensor muscle activation but without differences for any sitting parameter.

**Electronic supplementary material:**

The online version of this article (10.1186/s12891-018-2387-x) contains supplementary material, which is available to authorized users.

## Background

Many clinicians use movement-related interventions to treat low back pain (LBP) based on a view that there is a relationship between back pain and dysfunctional movement. There is some evidence that interventions designed to modify movement behaviour are associated with improvements to pain and activity limitation in chronic LBP [1, 2]. However, these studies have typically quantified changes to pain and activity limitation but not changes to movement qualities, so the relationship between change in movement behaviour and changes in pain and function is not clear.

The movement qualities of people with LBP have been observed to differ from those without LBP in a number of ways, including smaller range and lower speed of lumbar motion [3], differences in muscle size, recruitment and relaxation patterns [4–8], different breathing patterns [9–12], poorer proprioception [13–15], less motor control variability [16–20], poorer strength, endurance and muscle force control [21, 22] and different patterns of flexion-related lumbo-pelvic movement [23]. Although there is evidence of different movement qualities in people with back pain, there is little consensus about which movement attributes are important, how frequently they are seen, or whether movement difference might cause, or be caused by, LBP.

Recent movement research has mostly used some type of opto-electronic measurement, often in a laboratory setting, however wireless inertial motion and electromyography sensors that measure movement are now available and practical for use in both clinical and every-day-life settings. Inertial motion sensors are capable of providing detailed, precise kinematic information that is not easily measured by visual observation or through basic measurement tools, such as goniometers or flexible rulers. This ‘higher definition’ information provides a detailed picture of the magnitude, regional contributions and ‘quality’ of movement. Kinematic parameters such as relative range of movement (ROM) of body regions (e.g. lumbar spine versus pelvic movement), symmetry of ROM, movement speed, sequencing and timing of regional contributions (i.e. do lumbar and pelvic contributions move synchronously), and pelvic tilt kinematics (such as tilt angles, range from full anterior to full posterior tilt, trunk versus pelvic movement during tilting) can be combined with surface electromyographic (sEMG) information about lumbar or other muscle activation during movement. However, the clinical relevance of such kinematic parameters remains unclear. If kinematic parameters have a relationship to LBP, causal or consequential, they should be more prevalent in people with LBP than in those without LBP, even if not all people with LBP have the same movement characteristics.

A common clinical practice is to identify movement that is painful and/or ‘atypical’. A simple example would be to classify atypical ROM by identifying people whose ROM is particularly small or large, relative to a population without back pain. A similar process of classifying movement as atypical could be applied to movement timing, lumbo-pelvic rhythm (e.g. the sequence and pattern of lumbar versus pelvic contribution to movement during flexion) and muscle activation parameters. Exploratory analysis of detailed kinematic assessment and the prevalence of atypical movement may provide empirical evidence to inform and clarify the clinical practice of attempting to differentiate atypical from normal movement.

This exploratory study had four aims:

1. To ***describe*** the lumbo-pelvic kinematic parameters that can be measured with wearable inertial motion sensors, when investigated in two clinically-relevant types of lumbo-pelvic function: flexion (assessed during standing forward bending) and sitting.
2. To ***explore and define*** criteria that could classify these kinematic parameters as typical or atypical.
3. To ***investigate*** and ***compare*** the prevalence of atypical kinematic parameters in people with LBP (LBP group), and people who have never had back pain (NoLBP group).
4. To examine the relationship between atypical kinematic parameters and pain reported during standing forward bending or sitting activities.

We limited this initial, exploratory investigation to the analysis of flexion and sitting kinematic parameters only, to develop and test a method for classifying movement as atypical, and to compare the prevalence of atypical kinematic parameters in people with and without LBP. Lumbo-pelvic flexion has a relatively large range of motion compared to other physiological movements, has kinematic parameters of timing and sequence that are of potential clinical interest, and is often implicated as problematic in functional activities such as bending and lifting. Sitting kinematics were also included because sitting is often associated with LBP and because there is a belief that sitting posture is associated with LBP [24].

As we do not have a clear understanding of what represents atypical movement, this study was exploratory and descriptive, without pre-specified hypotheses.

## Method

### Study design and selection: Inclusion and exclusion criteria

We used an observational, cross-sectional design for this exploratory study. Participants with current back ± leg pain (LBP) were recruited using poster and word-of-mouth advertising from three Australian physiotherapy clinics/outpatient departments in primary and secondary care in 2014–2107. They were also recruited during 2011 at the Medical Department of the Spine Centre of Southern Denmark, which is an outpatient secondary care hospital department. All participants were measured at the site of recruitment. The inclusion and exclusion criteria, recruitment strategies, measurement protocols and test procedures have previously been reported in detail [25, 26] for the Australian sample and the same procedures were used in the Danish sample. Ethics approval was obtained from Monash University Human Research Ethics Committee (approval number 2016–1100) and from The Regional Committees on Health Research Ethics for Southern Denmark (approval number S-20110071). All participants gave written informed consent.

### Measurement protocol and test procedures

**Fig. 1:**
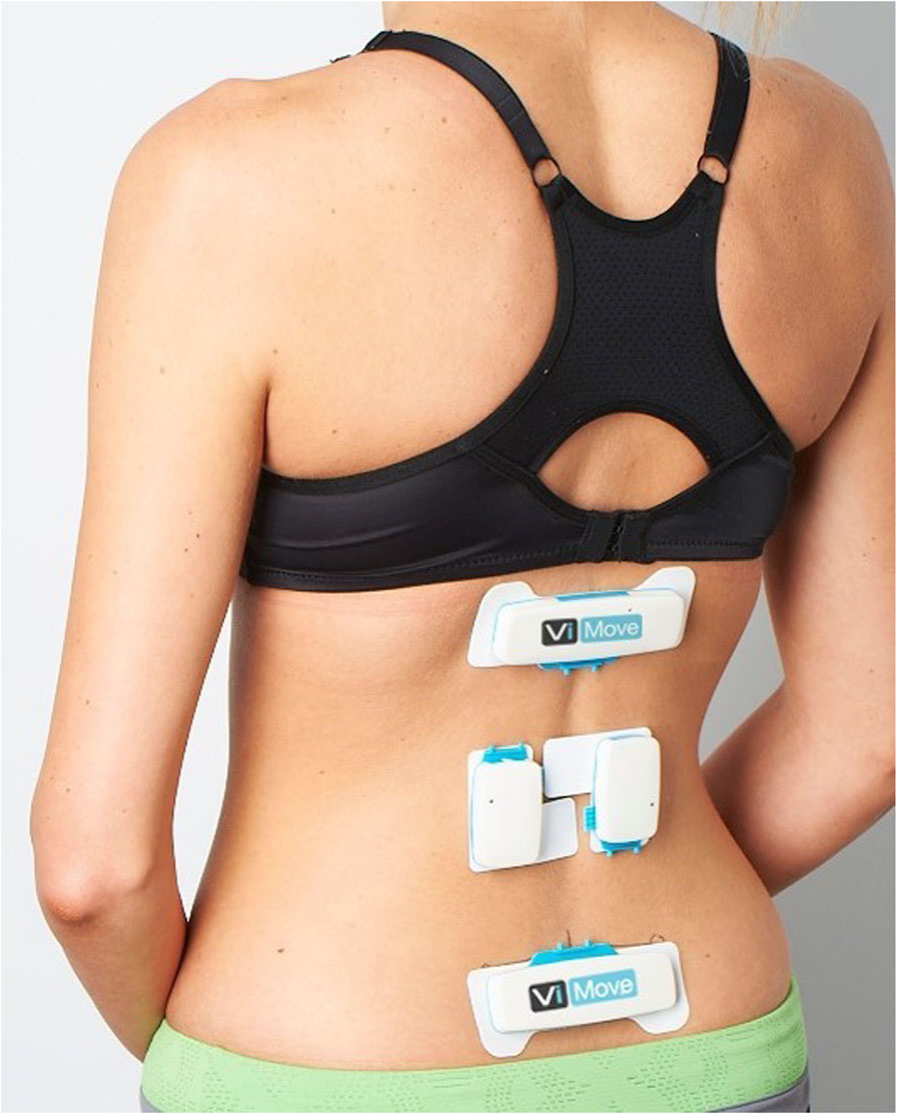
**Device Placement**. An example of sensor placement with the lower border of the upper sensor placed at the T12 level, the upper border of the lower sensor level with S1 and the EMG sensors placed over lumbar extensor muscles at the level of L3

Each participant completed an 11 point numerical pain rating scale (scores 0–10 where 10 = maximum pain intensity) [27], a 24 question Roland Morris Disability Questionnaire (RMDQ-24) [28] scored as a percentage with 100% = maximum activity limitation [29] and a specifically designed questionnaire about direction-specific pain (described fully in Laird et al. [26] and also available as Additional file 1: Appendix 1) prior to testing. All participants attended a single test session, where they were partially undressed to expose the body from T12 to the posterior superior iliac spines (PSIS). Shoes were removed. Two inertial motion sensors were then applied at T12 and S2 using adhesive backings and two surface electromyography (EMG) sensors were placed 1.5 cm either side of the L3 spinous process (see Fig. 1). A patient-height adjusted, plastic template was used to assist placement. A standardized testing procedure, including palpation of bony landmarks, device application and verbal instruction, was performed by six trained physiotherapists and three final year physiotherapy students, all of whom had received at least three hours specific training to minimize differences between testers. Reliability data has previously been published [25, 30]. With each participant, a single practice of the standing flexion movement was initially performed to test that sensors were working correctly and to ensure correct calibration. Subsequently, a minimum of three flexion repetitions were performed. The participant stood in a comfortable position and was instructed to bend forwards to the fully flexed position at their natural speed and hold this position for three seconds period using a counted time signal before return to upright standing. They then assumed three sitting postures, usual, upright and slumped, each for 15 s, with data captured in the last 5 s period. Lastly, while still sitting, they performed three repetitions of pelvic tilt. Testing protocols and movements can be viewed in Additional file 2: Appendix 2. All kinematic data were automatically captured at 20 Hz by the ViMove system (Dorsavi, Australia), independently of the assessor, and exported from the ViMove software as raw data, along with a system-generated graphic representation of data.

### Details and definition of kinematic characteristics

Eleven flexion and three sitting kinematic parameters were selected a priori for assessment (summarised in Table 1) and described in detail in the subsequent text.

**Table 1:** Summary of lumbo-pelvic kinematic parameters assessed

	Measurement Units
Standing flexion kinematic parameters	
Trunk angular inclination at T12 (upper motion sensor)	Degrees
Pelvic angular inclination at S2 (lower motion sensor	Degrees
Lumbar range of motion (difference between T12 and S2 sensors)	Degrees
Lumbo-pelvic coordination (rhythm) – peak angle, lumbar percentage	Percentage
Lumbo-pelvic coordination (rhythm) – across all movement, lumbar percentage	Percentage
Flexion relaxation response	Ratio
Delay (lag) of pelvic or lumbar movement at onset	Time (seconds)
Delay (lag) of pelvic or lumbar movement at 20^o^ of angular inclination	Time (seconds)
Delay (lag) of pelvic or lumbar movement at 30^o^ of angular inclination	Time (seconds)
Delay (lag) of pelvic or lumbar movement at 40^o^ of angular inclination	Time (seconds)
Duration of flexion movement (from standing to full flexion)	Time (seconds)
Sitting kinematic parameters	
Sitting pelvic tilt angular inclination range at S2	Degrees
Pelvic tilt ratio (maximum S2 movement/maximum T12 movement)	ratio
‘Relative’ lumbar ROM in sitting	Degrees

### Range of motion (ROM)

Trunk ROM was measured as angular inclination of the trunk at T12, pelvic ROM was measured as angular inclination of the pelvis at S2 and lumbar ROM was calculated using the difference between the angular inclinations at T12 and S2.

### Lumbo-pelvic coordination (rhythm)

Lumbo-pelvic coordination, sometimes described as lumbo-pelvic rhythm, is a method of describing lumbar versus pelvic contributions to movement. We calculated the relative contribution of lumbar movement and compared two methods (i) using peak angles at the end range of trunk flexion by using lumbar peak angle divided by trunk peak angle and expressed as a percentage, and (ii) using ‘area-under-the-curve’ method which sums all lumbar ROM and all pelvic angular inclination data at 20 samples per second from the start of flexion to a return to standing.

### Flexion relaxation response (FRR)

**Fig. 2:**
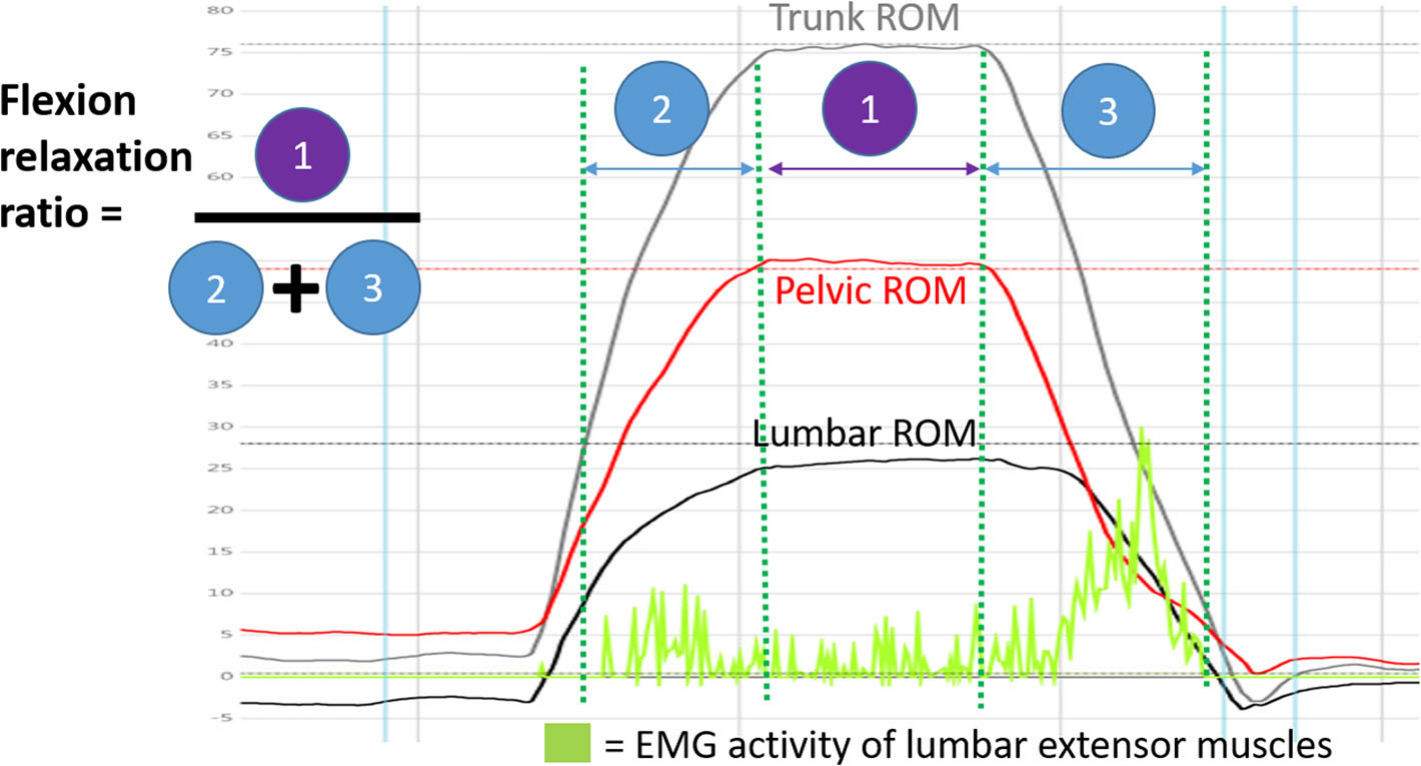
Flexion relaxation ratio definition and calculation. The flexion relaxation ratio is calculated by dividing EMG activity while the subject is fully flexed for 3 s (numerator) by the sum of EMG activity in the eccentric plus concentric phases of flexion (denominator)

A common pattern of thoraco-lumbar extensor muscle activity measured by surface electromyography (sEMG) is seen in people without back pain with electrical activity occurring at the start of trunk flexion (eccentric activation) and again on return from the fully flexed position (concentric activity), with minimal or no activity in the fully flexed position. This has been described as the flexion relaxation response (FRR) [31]. Flexion relaxation is often absent in people with LBP when compared to people without LBP, and when restored, is associated with improvements in pain and activity limitation [32, 33]. It is possible that higher extensor muscle activity in the fully flexed position, a position that is recognized as a biomechanically vulnerable position for the intervertebral disc [34], increases compressive loading. This study calculated the FRR ratio (following published methods [35–37]) using the sum of sEMG activity (millivolts) during 3 s in the fully flexed position (numerator) divided by the summed sEMG activity during both the eccentric (forward bending) and concentric (returning to upright stance) phases of flexion (denominator), (see Fig. 2). The ‘normal’ complete muscle relaxation in full flexion would result in the FRR being close to or equal to zero. Any muscle activity during end-range flexion increases this ratio, with a larger number indicating greater muscle activation and reduced relaxation in the fully flexed position. Raw sEMG activity (microvolts) was sampled at 300 Hz, then a high pass filtering was applied using a ‘fast fourier transformation’ algorithm. A low pass filtering occurred to create an envelope of the signal at 20 Hz. Finally, the signal was transformed using a root-mean-square (RMS) process to measure muscle activity.

### ‘Delay’ (lag) between pelvic and lumbar movement

Because motion sensors measure movement over time, it is possible to assess time-related synchronicity of lumbar versus pelvic contributions to flexion movement. There is evidence of time-related differences in lumbar versus pelvic movement during flexion [38]. An ‘onset-delay’ parameter measures which region, lumbar or pelvis, moves first and the time ‘gap’ between regions. Negative numbers indicate a delay in pelvic motion, with movement initiated first in the lumbar spine, while positive numbers indicate a delay in lumbar motion, with movement initiated at the pelvis. Larger numbers indicate a longer delay. The start of flexion was defined as the point at which velocity was >7^o^/sec (the velocity required before movement was visible graphically).

**Fig. 3:**
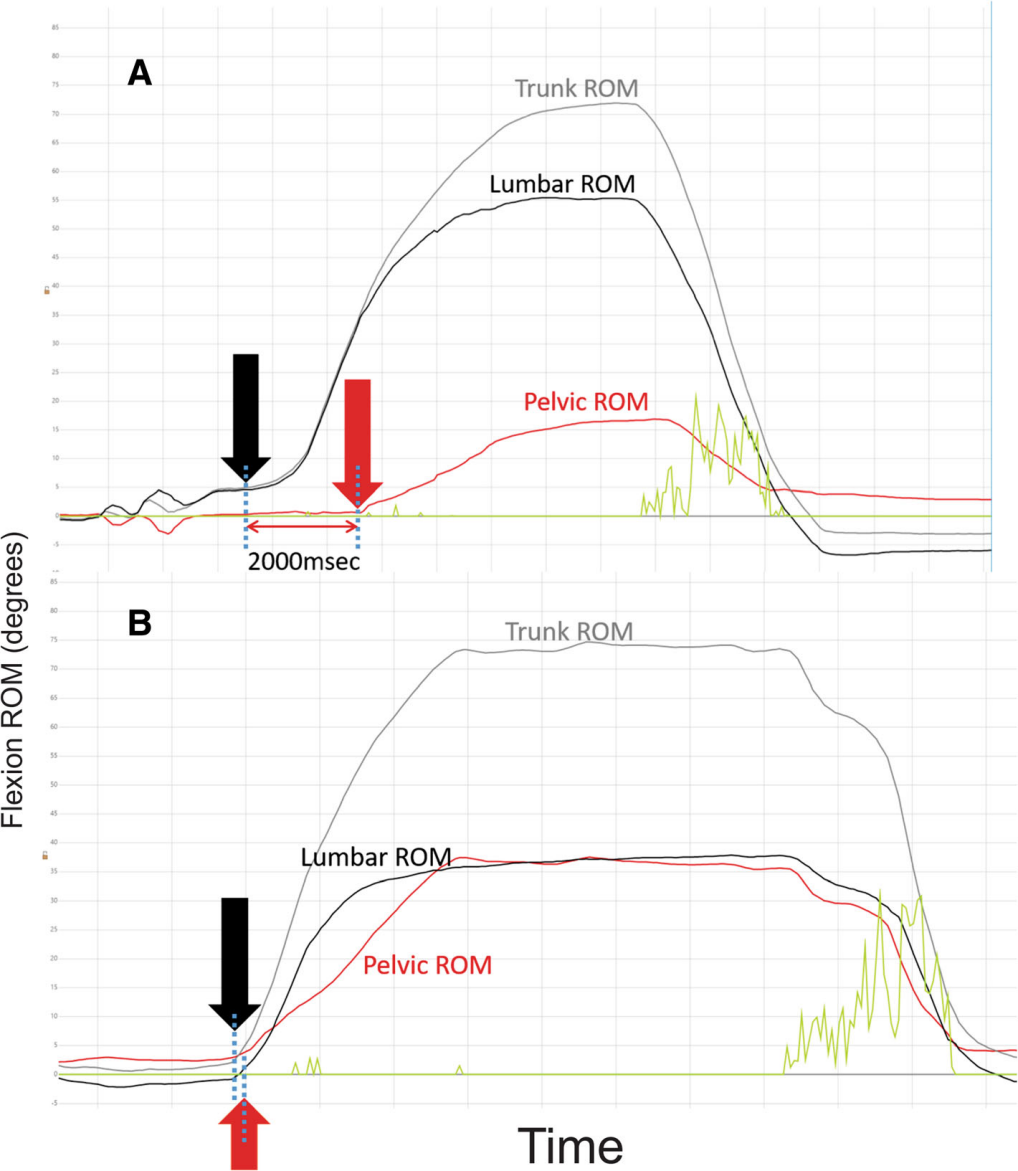
Delay (lag) of pelvic compared to lumbar movement. These graphs show ROM (Y axis) changes over time (X axis). Graph **a** was from a subject who moved their lumbar spine into flexion with a two second delay before the pelvis started moving. Graph **b** shows a more typical pattern with a synchronous start of movement of the lumbar spine and pelvis

Figure 3 demonstrates an example of an onset-delay in pelvic movement. The ‘delay-at 20^o^, 30^o^ and 40^o^’ parameters provide a similar view of movement discrepancy and is a calculation of the time needed to achieve 20^o^, 30^o^ and 40^o^ of angular inclination from the start of movement, for each region. These parameters provide a measure of time-related synchronicity (or lack thereof) of lumbar versus pelvic contribution to flexion.

### Flexion movement duration

Flexion movement duration was defined as the time taken from start of trunk flexion (when velocity of movement was > 7°/sec) to the fully flexed position (when velocity was < 7°/sec velocity). We defined end of trunk flexion in this way because movement with a velocity less than 7^o^/sec is very close to end-range and this threshold minimizes error that can result from the peak angle slowly increasing due to creep when the fully flexed position is sustained for the three second period during which we assessed the flexion relaxation response.

### Sitting: Pelvic tilt range and pelvic tilt ratio

Pelvic tilt ROM (from full posterior to anterior tilt angular inclination) may be of clinical interest when sitting is associated with pain. Reduced pelvic repositioning accuracy (proprioception) and reduced movement variability have been identified in people with chronic LBP [3, 17, 19, 20, 39]. The pelvic tilt range was measured by calculating the angular inclination of the pelvis between full anterior and full posterior tilt, which provided estimates of lower lumbar movement. The pelvic tilt ratio is a measure of the independence of pelvic tilt relative to trunk movement and is calculated by dividing the angular inclination of the pelvic sensor by the angular inclination of the trunk sensor. This parameter was used to test how pelvic tilting was performed i.e. whether movement was independently performed with mostly lower lumbar motion or combined with upper lumbar motion, as might occur if a subject simultaneously moved the trunk into flexion while performing posterior pelvic tilt). A number > 1 indicates larger pelvic than trunk ROM; a number < 1 indicates larger trunk than pelvic ROM during the pelvic tilt manoeuvre.

### Sitting: Relative position

Measurements were made of usual, full slumped (kyphotic) and full upright (lordotic) sitting lumbar positions. The relative sitting position was calculated for usual sitting by deeming the fully slumped sitting position to be 100% and the fully upright sitting to be 0%. For example, if full slump was at 50^o^ of lumbar flexion and full upright sitting was at 0^o^ lumbar flexion, then the difference (50^o^-0^o^ = 50^o^) between maximum slump and upright sitting would represent 100% of the available ROM. If usual sitting was 25^o^, the relative sitting position would have been coded as 50%. This index enabled comparisons between individuals for defining usual sitting position relative to the available range of pelvic movement.

### Pain scores for bending and sitting activity

In addition to a numerical rating scale for pain, people with back pain were asked, using a self-completed, non-validated questionnaire “Is your pain aggravated by bending forwards activities?”, scored as (0) never, (1) rarely (2) sometimes, (3) often, (4) always and then a further multiple choice based on the level of pain aggravation: (0) none, (1) low, (2) medium, (3) high. An overall score was calculated by multiplying the two answers to give scores ranging from 0 to 12. We used this method, despite having only face validity, as it reflects the common clinical practice of establishing the severity and frequency of pain associated with aggravating activities. Scores were then arbitrarily dichotomized a priori, into < 6 or 6 or greater. Similarly, a ‘pain on sitting’ score was derived by asking “How long can you sit before feel you have to stand up?” (< 5 min, 10, 20, 30, 60 or > 60 min), scored 5–0 and “If pain stops you from sitting any longer, what is your level of pain?” on a scale of 0–10. A total score for sitting was calculated by multiplying the two sitting scores for a maximum score of 50 which was then dichotomized to 18 or greater based on the arbitrary choice of the median score.

### Equipment

The ViMove system, version 5, (DorsaVi, Australia) is an inertial measurement system comprised of two wireless movement sensors containing a triaxial accelerometer, a triaxial gyroscope and a magnetometer, two wireless surface EMG sensors, and a small wireless recording device that can be easily carried (e.g. in a pocket). Average differences of < 2° have been reported for through-range flexion movements when compared to a VICON opto-electronic device [40]. The ViMove version 5 movement sensors collected data at 20 Hz.

### Sample size

As this was an exploratory study, no data were available for sample size calculations. The aim was to test a sample large enough to enable the development of hypotheses but not so large as to waste resources should there be no interpretable findings. Samples of over 100 per comparison group were considered large enough to indicate the likelihood of observable patterns in the data and provide insight into sample sizes required to test hypotheses arising from this work. As subjects in both Australia and Denmark were assessed using the same procedures, their data were pooled to maximise data available for analysis.

### Data analysis

Data were analysed from Danish data collected during 2011 using version 4.5 of the ViMove software and Australian data collected between 2014 to 2017 using version 5.10. The software version did not affect the method of data collection or accuracy. Movement data were exported from the ViMove software into Excel spreadsheets (Microsoft Corp, Redmond, WA USA) for data cleaning and graphical visualisation (for an example, see Figs. 2 and 3). Each data capture was visually checked for accuracy and the first three repetitions of each movement were averaged to improve consistency.

### Statistical analysis

Movement data were analysed using multivariable linear regression to examine the effects of group (LBP, noLBP), with age and gender included as co-variates (controlled) in the model. We reported the absolute size of the score on each movement parameter for the LBP and noLBP, and the adjusted difference between those scores (the beta coefficient from the regression model) and its *p*-value. Where that *p*-value was < 0.05, we reported the actual value, otherwise simply reported it as not significant (NS).

Each kinematic characteristic was then dichotomized into atypical or typical using the arbitrary cut-point of the 10th centile value derived from the NoLBP group. For each parameter, the lowest 10% of values in the NoLBP group was classified as atypically small and the remaining 90% of values classified as typical. A similar logic was applied in interpreting the highest 10% of values as atypically large. The frequency of atypical movement was then reported for each group. As age and gender are known to be associated with range of movement [3, 41], age and gender adjusted prevalence ratios were calculated using logistic regression, with the resultant odds ratios being converted into prevalence ratios using the STATA oddsrisk command. Dichotomised pain scores for ‘high-pain-on-bending’ and ‘high-pain-on-sitting’ were tested for their association with atypical kinematic variables using logistic regression. STATA 14.0 was used for all statistical analysis (StataCorp, College Station TX, USA).

## Results

### Demographics

Participant gender and age, are presented in Table 2. There were 24 NoLBP and 35 LBP Danish subjects. There was no difference in age, gender or BMI between Australian and Danish subjects. For the LBP group, the mean pain score (and standard deviations) on a 0–10 scale was 5.3 (1.5) and activity limitation (RMDQ-24 transformed to a 0–100 scale) was 39 (21). There was a significant difference in age, with people with LBP being, on average, 7 years older than people with no back pain. However, on all the movement parameters, there were no statistically significant associations between the prevalence of atypical movement in the LBP and NoLBP groups and either age or gender. This was also reflected by the unadjusted and adjusted (age and gender) prevalence ratios being almost identical (data not shown).

**Table 2:** Demographics

	N (for ROM, LPR and FRR)*	N (for time-related and sitting data)	Age (mean ± SD)	Gender
NoLBP	126	100	34.4 ± 13.5**	41% Male
LBP	140	105	41.4 ± 12.6**	43% Male

*ROM range of motion*, LPR* lumbo-pelvic rhythm*, FRR* flexion relaxation response

** p = .0001

Due to software version evolution between 2011 and 2014, time related and sitting data could only be analysed for data collected after 2014 (LBP group = 105 and NoLBP = 100). The range of movement related data, including lumbo-pelvic rhythm and flexion relaxation response, were available for all participants.

### Flexion kinematic data

Between group comparisons (mean, standard deviations, 10th and 90th percentiles) for all flexion kinematic data are reported in Tables 2 and 3.

**Table 3:** Range of movement, lumbo-pelvic rhythm and FRR parameters

Movement parameter	Details	No LBP (*n* = 124)	LBP (*n* = 140)	*p*-value
Peak trunk flexion	Trunk flexion angular inclination (T12)	111^o^ ± 16^o^	93^o^ ± 16^o^	*p* < .0001 **β = − 16 (− 20, − 12)
Small trunk ROM (10th centile, <93^o^)	Number (%) of people with small trunk flexion	11 (10%)	67 (47.8%)	*p* < .0001
	Prevalence ratio*	–	5.4 (3.5–7.3)	
Large trunk ROM (90th centile, > 128^o^)	Number (%) of people with large trunk flexion	12 (10%)	4 (3%)	*p* = .008
	Prevalence ratio	–	0.3 (0.1–0.9)	
Peak lumbar flexion	Lumbar ROM	52^o^ ± 11^o^	46^o^ ± 12^o^	*p* < .0001 β = − 6 (− 9, − 12)
Small lumbar ROM (10th centile, <39^o^)	Number (%) of people with small lumbar flexion	12 (10%)	41 (29.3%)	*P* = .0001
	Prevalence ratio	–	3.0 (1.8–4.7)	
Large lumbar ROM (90th centile, >65^o^)	Number (%) of people with large lumbar flexion	13 (10%)	8 (6%)	NS
	Prevalence ratio	–	0.5 (0.2–1.2)	
Peak pelvic flexion	Pelvic flexion angular inclination (S2)	59^o^ ± 15^o^	48^o^ ± 15^o^	*p* < .0001 β = −11 (−14, −7)
Small pelvic ROM (10th centile, <42^o^)	Number (%) of people with small pelvic flexion	10 (9%)	48 (34%)	*p* < .0001
	Prevalence ratio	–	3.9 (2.3–5.8)	
Large pelvic ROM (90th centile, >75^o^)	Number (%) of people with large pelvic flexion	13 (10%)	7 (5%)	NS
	Prevalence ratio	–	0.5 (0.2–1.1)	
Lumbo-pelvic co-ordination	Mean Lumbar % contribution	48 ± 11%	49 ± 11%	NS β = 1.8 (1, 5)
Small Lx contribution (10th centile, < 38%)	Number (%) of people with small lumbar contribution	13 (10%)	19 (14%)	NS
	Prevalence ratio	–	1.3 (0.7–2.4)	
Large Lx contribution (90th centile, > 63%)	Number (%) of people with large lumbar contribution	11 (9%)	18 (13%)	NS
	Prevalence ratio	–	1.5 (0.7–2.8)	
FRR	Means units of surface EMG activity	0.012 ± 0.32	0.25 ± 0.32	*p* < .0001 β = 0.24 (0.15, −0.31)
Low FRR (10th centile, > 0.033 units of EMG activity)	Number (%) of people with reduced FRR	13 (9%)	71 (52%)	*p* < .0001
	Prevalence ratio	–	4.9 (3.4–6.4)	

* Adjusted prevalence ratio’s considering the effect of age and gender are reported only, as there was minimal difference between unadjusted and adjusted ratios indicating minimal effect of age and gender

**β = the beta coefficient (and 95% confidence intervals) from regression models, which represents the size of the difference between the two groups, adjusted for age and gender

#### Peak angular data

Significant mean (SD) differences between the NoLBP and LBP groups were found for trunk peak angle (NoLBP 111^o^ *(16*^*o*^*); LBP 93*^*o*^ *(16*^*o*^*), p < .0001*), lumbar peak angle (NoLBP 52^o^ *(11*^*o*^*); LBP 46*^*o*^ *(12*^*o*^*), p < .0001*) and pelvic peak angle (NoLBP 59^o^ *(15*^*o*^*); LBP 48*^*o*^*, (15*^*o*^*), p < .0001*) (Table 3). People with a small ROM were 5.4 (95% CI 3.0–9.7, *p* < .0001) times more prevalent in the LBP group for trunk ROM when adjusted for age and gender differences. Similar values were seen for lumbar and pelvic ROM (Table 2). There was no difference in the prevalence of atypically large ROM between groups for trunk, lumbar or pelvic angles (see Table 3).

#### Lumbo-pelvic rhythm (LPR)

There were no differences between groups for the percentage of lumbar (versus pelvic) contribution to overall trunk flexion movements, and minimal, non-significant differences in prevalence rates when both low and high lumbar percentage contribution were compared (Table 2) when using both peak angle and area-under-the curve methods. There was no difference in the results from the two methods of calculating the percentage of lumbar contribution, so the less complex approach of peak angle was reported and the more complex calculation method using the area-under-the-curve approach was dropped from further reporting.

#### Flexion relaxation response (FRR)

Significant differences between the NoLBP and LBP groups were found for a low FRR ratio (NoLBP 0.012*, (0.32); LBP 0.25, (0.32), p < .0001*) indicating a greater loss of flexion relaxation in the fully flexed position for the LBP group. The prevalence of low FRR (greater activity of extensor muscle in the fully flexed position) was 4.9 (95% CI 2.9–8.4, *p* < .0001) times greater in the LBP group when compared to the NoLBP group (Table 4).

**Table 4:** Timing and sitting parameters

Movement parameter	Details	No LBP (*n* = 100)	LBP (*n* = 105)	*p*-value
Delay at 0^o^	Mean delay (negative numbers indicate pelvic delay)	-0.21 ± 0.46 s	-0.36 ± 0.46 s	*p* = .023 **β = − 0.15 (− 0.28, − 0.21)
Pelvic delay at onset of movement (10th centile, > 0.53 s)	Number (%) of people with pelvic delay > 0.53 s	10 (10%)	19(18%)	NS
	*Prevalence ratio	–	2.0 (0.9–3.3)	
Lumbar delay at onset of movement (90th centile, > 0 s)	Number (%) of people with lumbar delay > 0 s	11 (11%)	10 (10%)	NS
	Prevalence ratio	–	1.1 (0.04–0.8)	
Delay at 20^o^	Mean delay (negative numbers indicate pelvic delay)	− 0.30 ± 0.88 s	−0.51 ± 0.90s	NS β = − 0.21 (− 0.46, 0.44)
Pelvic delay at 20^o^ of trunk flexion (10th centile, > 0.81 s	Number (%) of people with pelvic delay > 0.81 s	10 (10%)	29 (29%)	*p* = .0007
	Prevalence ratio		2.9 (1.6–4.7)	
Lumbar delay at 20^o^ of trunk flexion (90th centile, > 0.15 s)	Number (%) of people with lumbar delay >.15 s	9 (9%)	18 (18%)	NS
	Prevalence ratio		2 (0.9–3.8)	
Mean movement duration	Time from start of flexion to full flexion	2.28 ± 0.94	3.18 ± 0.94	*p* < .0000 β = 0.90 (0.64, 1.16)
Slow Trunk movement (10th centile, > 3.12 s)	Number (%) of people with Slow Trunk movement	10 (10%)	49 (47%)	*p* < .0000
	Prevalence ratio	–	4.7 (2.9–6.5)	
Mean pelvic tilt range	Range from full anterior tilt to full posterior tilt	29^o^ ± 13^o^	29^o^ ± 13^o^	NS β = −0.3 (−3.8, 3.3)
Small pelvic ROM (10th centile, < 11^o^)	Number (%) of people with small pelvic tilt range	10 (10%)	10 (10%)	NS
	Prevalence ratio	–	1.0 (0.4–2.2)	
Large pelvic ROM (90th centile, >49^o^)	Number (%) of people with large pelvic flexion	10 (10%)	6 (6%)	NS
	Prevalence ratio	–	0.6 (0.2–1.5)	
Mean pelvic tilt ratio	Pelvic tilt range/range of trunk ROM change	2.1 ± 1.3	2.4 ± 1.4	NS β = 0.4 (0, 0.7)
Small tilt ratio (10th centile, < 0.69)	Number (%) of people with small pelvic tilt range	10 (10%)	6 (5.7%)	NS
	Prevalence ratio		0.58 (0.2–1.5)	
Large tilt ratio (90th centile> 3.8)	Number (%) of people with large pelvic flexion	10 (10%)	13 (12%)	NS
	Prevalence ratio		1.27 (0.6–2.6)	
Mean relative sitting position	Max slump sit = 100%, maximum upright sit = 0%	48 ± 35%	50 ± 35%	NS β = 2 (−7, 12)
Slumped sitting (10th centile, > 89%)	Number (%) of people with slumped sitting	10 (10%)	16 (16%)	NS
	Prevalence ratio	–	1.7 (0.8–3.2)	
Upright sitting (90th centile, > 12%)	Number (%) of people with upright sitting	10 (10%)	10 (10%)	NS
	Prevalence ratio	–	1.0 (0.4–2.2)	

* Adjusted prevalence ratio’s considering the effect of age and gender are reported only, as there was minimal difference between unadjusted and adjusted ratios indicating minimal effect of age and gender

**β = the beta coefficient (and 95% confidence intervals) from regression models, which represents the size of the difference between the two groups, adjusted for age and gender

**Table 5:** Relationship of high pain score to kinematic parameters

Kinematic parameter	Total Number of LBP subjects with data	No. of LBP subjects with atypical movement	No. of LBP subjects with LOW PAIN score on bending/sitting	No. of LBP subjects with HIGH PAIN score on bending/sitting	Association with ‘HIGH PAIN on bending/sitting’ score
Flexion kinematic parameters					
Small Trunk ROM	135	64	27	37	NS
Large Trunk ROM	135	4	2	2	NS
Small Lumbar ROM^**a**^	135	38	12	26	*p* = .012
Large Lumbar ROM	135	7	2	5	NS
Small Pelvic ROM^**a**^	135	44	14	30	*p* = .011
Large Pelvic ROM	135	6	4	2	NS
Small LPC	135	1	9	8	NS
Large LPC	135	16	8	8	NS
Low FRR	132	67	33	34	NS
Pelvic delay at onset	101	17	10	7	NS
Lumbar delay at onset	101	16	10	6	NS
Pelvic delay at 20o	96	28	15	13	NS
Lumbar delay at 20o	96	19	10	6	NS
Slow trunk movement	101	47	26	21	NS
Sitting kinematic parameters					
Small Pelvic tilt range	100	9	6	3	NS
Large Pelvic tilt range	100	6	2	4	NS
Small tilt ratio	100	5	5	0	NS
Large tilt ratio	100	12	7	5	NS
Slumped sitting position	100	17	7	10	NS
Upright sitting position	100	9	5	4	NS

*ROM* range of motion, *FRR* flexion relaxation response, *LPC* lumbo-pelvic co-ordination, *NS* nonsignificant

^**a**^Significant difference with greater frequency of people reporting higher pain scores

#### Onset delay and at 20^o^ of trunk movement

The time difference comparing lumbar to pelvic movement reaching 20^o^ of angular inclination was reported, and the alternative computation of comparisons at 30^o^ and 40^o^ were dropped, as almost all participants produced a reading of 20^o^ for both lumbar and pelvic movement, whereas at 30^o^ and 40^o^, 13 and 33% of participants respectively did not achieve these angles for either lumbar or pelvic motion. Significant differences between the NoLBP and LBP groups were found for ‘onset-delay’, with a between group difference of greater delay in pelvic motion for the LBP group (NoLBP -0.21, (*0.46)sec; LBP -0.36, (0.46)sec, p = 0.023).* There were no significant differences in atypically delayed lumbar or pelvic movement at onset. Atypical ‘delay-at 20^o^’ for pelvic movement was significantly more prevalent (2.9 times) in the LBP group (95% CI 1.5–5.6, *p* = .0007) (Table 4).

#### Flexion movement duration

Significant differences between the NoLBP and LBP groups were found for flexion movement duration (NoLBP 2.28 (*0.94)sec; LBP 3.18 (0.94)sec, p < .0001*). The prevalence of atypically long flexion movement duration (slow trunk movement) was 4.7 (95% CI 2.5–8.7, *p* < .0001) times greater in the LBP group than for the NoLBP group (Table 4).

#### Sitting: Pelvic tilt range and relative sitting position

There were no differences found for pelvic tilt range, pelvic tilt ratio or for relative sitting position between groups. There were no between group differences in the prevalence of atypical sitting parameters (Table 4).

#### Relationship between pain scores and atypical flexion or sitting movement

There was a significantly greater frequency of higher pain scores on bending in people with small lumbar ROM or small pelvic ROM. No other flexion or sitting kinematic parameter demonstrated differences in the frequency of high pain scores between the NoLBP and LBP groups (Table 5). Five LBP subjects had incomplete pain scores and therefore were not included in that analysis.

## Discussion

### Brief summary of findings

This exploratory study measured flexion (in standing) and sitting lumbo-pelvic kinematic parameters, in typical clinical settings, using wearable wireless inertial motion sensors in people with and without LBP. We examined between-group differences, defined and calculated the prevalence of ‘atypical’ flexion and sitting kinematic parameters for each group, and tested the relationship between ‘high pain’ scores and atypical movement. Between group differences showed less trunk, lumbar and pelvic ROM, less flexion relaxation, delayed pelvic movement at the start of movement and slower trunk flexion for the LBP group. Using the 10th/90th centiles for people without LBP to establish atypical movement parameters, we found a significantly greater prevalence of small trunk, lumbar and pelvic ROM for the LBP group, but not for large trunk, lumbar or pelvic ROM. Similarly, there was a greater prevalence in the LBP group for less flexion relaxation, slow trunk movement and delayed timing of pelvic (versus lumbar) movement to achieve 20^o^. No between group differences were seen for lumbo-pelvic co-ordination or for any of the sitting parameters. For most atypical kinematic parameters, there was no relationship with high pain scores during flexion or sitting, with the exception of small lumbar and pelvic ROM being associated with a high score on pain on forward bending.

### Defining atypical movement with a dichotomising approach

Previous studies have reported similar between-group differences for lower ROM [3], slower movement velocity [3, 42, 43] and less flexion relaxation [32] in those with LBP. We used the term ‘atypical’ rather than dysfunctional or abnormal movement because movements that are atypical were present in both groups. Defining atypical movement and dichotomizing the data, allowed testing of the prevalence of both low ***and*** high values for each parameter. This was useful because both the LBP and NoLBP groups included people with atypically small ***and*** large values for all parameters. The presence of atypical movement in people without a history of significant LBP suggests that these parameters may pre-exist pain. However, the significantly higher prevalence of atypically small ROM, less flexion relaxation, longer movement duration and delayed pelvic movement, suggests a relationship with pain. The nature of this relationship, whether causative or a consequence of pain, is unclear.

We chose a dichotomizing approach because it reflects decision making used in clinical practice and has potential utility in determining which movement components might be a target of therapeutic intervention. The use of 10th centile criterion was an arbitrary decision, based partially on a consideration of our sample size. Larger centiles could have been chosen but, by definition, atypical movement would have been more common. Smaller centiles could also have been used but would have needed larger samples because of the smaller number of people classified as having atypical movement and the corresponding increase in the uncertainty of the statistical estimates.

As atypical movement is present in people who have never had LBP a potentially important question for future research would be to explore in longitudinal studies whether some atypical movements are prognostic indicators for the development of LBP in some people.

### Rom

The results from our study indicate a significant relationship between the presence of LBP and small ROM, suggesting that identifying atypically low ROM maybe potentially important clinically. There is evidence of an association between pain-related fear, reduced ROM and poor flexion relaxation that is consistent with our data [44]. Assessing spinal movement in people with LBP has been problematic with large variations in reported lumbar ROM, poor reliability arising from differing measuring techniques and devices, and conflicting reports about the utility of measuring spinal movements as a measure of activity limitation [3, 45–49]. Nevertheless, ROM remains a common feature of assessing and monitoring musculoskeletal injury, suggesting that measuring ROM is still considered to have clinical importance. People with acute LBP often demonstrate a reduced ROM that returns to ‘normal’ as pain reduces, suggesting pain as a cause of small ROM. However, the presence of small ROM in the NoLBP population indicates that small ROM is not only a response to injury or pain, but maybe present prior to pain occurring. This has implications for monitoring ROM as a ‘response to change’ variable. For a person who had small ROM prior to injury, improvements in pain or disability may not be similarly associated with changes in ROM associated with recovery compared to a person who, prior to injury, had a large ROM. This factor might partly account for the limited association reported between pain, activity limitation and ROM [47]. It would also be easy to think of the LBP group as ‘restricted or stiffer’ (smaller ROM) than the NoLBP group, and while this appeared true for 48% of the LBP group, there was still considerable overlap with the NoLBP population. Indeed, some people with LBP have atypically high trunk, lumbar and/or pelvic ROM. While small ROM deficits are present in some people with LBP, they are not present in all LBP patients. So, interventions designed to improve or restore typical movement range are unlikely to be helpful if no, or minimal loss, of movement is present.

The concept of measuring both lumbar and pelvic ROM contributions to overall trunk flexion is not novel, however in a recent systematic review 10 out of 16 studies that measured flexion ROM only reported lumbar ROM [3]. Functional activities that involve trunk flexion include lumbar and pelvic motion. Our results indicate that atypically small pelvic ROM is significantly more prevalent in the LBP group, suggesting that pelvic ROM should also be measured when examining trunk flexion. For example, when assessing a person with back pain, typical lumbar ROM may be present but accompanied by atypically small pelvic ROM.

### Flexion relaxation and timing parameters

The absence of flexion relaxation has been repeatedly identified in people with LBP. Improvements to pain have been associated with improved flexion relaxation following interventions specifically aimed at reducing muscle activation of lumbar extensor muscles in the fully flexed position [37, 50, 51]. People with normal relaxation have a ratio near zero, so all ratio scores over 0.033 are atypically high ratio scores that indicate low/reduced flexion relaxation. Targeting people with LBP who have poor flexion relaxation is likely to be important, but not all people with LBP have poor flexion relaxation.

The clinical utility of the timing parameters measured with tools that can accurately measure movement over time is unclear. While it is biomechanically plausible that a relative delay or lag in pelvic or lumbar movement may have potential clinical implications by increasing biomechanical forces on upper or lower lumbar structures, there is currently no research evidence to support the clinical relevance of such findings. However, the observation that these delays exist and are more commonly seen in people with back pain suggests that they may have clinical relevance, but this requires further investigation. It is also plausible that slower movement velocity is a consequence of LBP and might be useful as a measure of change but there is no current evidence that slow movement may cause LBP.

### Patterns of atypical movement

People with LBP are frequently considered to be heterogenous in a range of domains such as differing cognitive perspectives, trajectories of improvement, movement patterns and patho-anatomical diagnoses [52–55]. Our data demonstrates a wide spectrum for most kinematic parameters for both groups, highlighting the heterogenous nature of movement. In this sample, people with LBP could equally have high or a low percentage lumbar contribution (lumbo-pelvic co-ordination) to overall flexion, which represent different methods of achieving trunk flexion. Similarly, different patterns in movement timing were seen in ‘onset-delay’ i.e. in which region moves first. A pelvic delay (indicating lumbar spine moving first) was twice as prevalent in the LBP group, while a lumbar delay was seen equally in both groups. The relative time for pelvic and lumbar components to achieve 20^o^ of flexion similarly reflected two different patterns of movement, where 29% of the LBP group had atypical, delayed pelvic movement and 18% had atypical lumbar delay. Overall, given the heterogeneity of these kinematic parameters, if a movement or position was associated with pain, and then targeted with a movement-based intervention, it is unlikely that a ‘one-size fits all’ approach will be helpful and that an individually targeted approach may be more likely to achieve better overall outcomes.

### The relationship of pain to atypical flexion and sitting parameters

Evidence for a relationship between pain and movement has been unclear. We expected that high pain on bending or sitting might have been associated with corresponding atypical kinematic parameters at either end of the spectrum (high or low values). Our results did support a relationship between ‘high-pain-on-bending’ scores with small lumbar and pelvic ROM, consistent with other studies [38, 44, 56] but not with other flexion-related parameters. There was no significant relationship between ‘high-pain-on sitting’ scores and any sitting kinematic parameters. Given that sitting is frequently listed as an aggravating activity in people with LBP and that sitting postures are thought to be associated with LBP [24] it would be reasonable to think that atypical end-range sitting postures might be associated with higher levels of pain, however this was not seen in this sample. People with LBP sat with large variation in position with 16% sitting in atypically slumped and 10% in an atypically upright position. There is some evidence that bio-feedback to modify end-range sitting positions reduces LBP [2] however further research is required to clarify the relationship of movement change to pain reduction.

The absence of a clear and consistent relationship between pain intensity and atypical movement might occur because pain is a multifactorial experience with numerous cognitive [57, 58], physiological and mechanical components, and does not necessarily have a linear correlation to activity limitation or participation restriction [59]. While it could be argued that pain may not be related to atypical movement, a number of trials of treatments that aim to modify movement in people with chronic LBP have shown improvements to pain and activity limitation [1, 60]. What is not known, but would be very useful to know, is whether those improvements in pain and activity limitation were mediated by changes in movement, or whether movement interventions improved those outcomes via other effects, such as increasing a sense of self efficacy or changing pain cognitions.

## Strengths

While numerous studies have reported lumbosacral ROM, this paper is different in that it dichotomizes movement into typical and atypical values. It highlights the utility of capturing a number of ‘high definition’ kinematic parameters that include regional movement, timing, sequence patterns and electrical activity, and defining atypical movement. Because data for both NoLBP and LBP groups was taken from a number of clinics and geographic locations, it is likely that data is representative of both groups, increasing the validity of generalising these results to the broader population. The sample size was relatively large for a kinematic study and therefore it is more likely that less commonly seen variants would be included in this sample. The precision of the measurements is high, with accuracy levels reported by the manufacturer of < 1° for single plane movement and good concurrent validity (< 2°) when comparing these wireless inertial sensors to other ‘reference-standard’ surface measurement systems [40, 61, 62]. The reported data has clinical utility with the dichotomous approach reflecting aspects of clinical practice and the chosen kinematic parameters based on potentially clinically important movement characteristics.

## Limitations

Skin surface measurement should be used cautiously as a representation of actual spinal movement, however it can be used to measure baseline and change characteristics, and to provide comparison between typical and atypical movement. Using a skin surface measurement technique to measure movement has the advantage of being non-invasive and possible within a typical clinical setting. While skin movement can create artefact, flexion is less exposed to this risk than other movements such as extension [25].

Sitting kinematics recorded as ‘usual, slumped and upright’ may not reflect real world sitting practice. The nature of real-world sitting, such as sitting in a car, or on the participant’s usual chair may alter the intensity or frequency of pain, as parameters of duration and sitting frequency were not explored in this study and are potentially important.

Higher prevalence rates of some atypical movement parameters may indicate an association with back pain, but low prevalence rates do not necessarily imply no relationship. It may be that some parameters such as high ROM are rarer, but are still related to back pain.

This study examines univariate relationships only. It is possible that multivariate relationships (patterns or clusters) may exist where variables combine in clinically relevant groups. Further research will examine these possibilities. Theoretically, it is feasible that there are subgroups of people with relatively mutually-exclusive clusters or patterns of atypical movement that relate to pain or activity limitation, or to factors from psycho-social dimensions of LBP. Future research could also include other physiological movements such extension, lateral flexion and rotation but were omitted from this paper to reduce complexity, and to allow a focus on exploring and developing atypical movement definitions.

## Conclusion

This exploratory, cross-sectional study used wireless inertial and EMG sensors to measure lumbo-pelvic kinematics during trunk flexion and sitting position (ROM, timing, sequence coordination, relative sitting position, pelvic tilt range and extensor muscle activation) in a sample of NoLBP and LBP subjects. For flexion, significant mean differences were seen with the LBP group demonstrating lower ROM, less flexion relaxation, a greater delay of pelvic movement at the onset of trunk movement and slower trunk flexion. Atypical movement was defined based on the 10th/90th centiles of the NoLBP group. People in the LBP group had a significantly greater prevalence of small trunk, lumbar and pelvic ROM, reduced FRR, slow trunk movement and delayed timing of pelvic (versus lumbar) movement to achieve 20° of angular inclination. No between group differences or prevalence rates were seen for large ROM, lumbo-pelvic co-ordination or for any of the sitting parameters. There was a relationship with high pain scores during flexion or on small lumbar and pelvic ROM but not with other flexion or any sitting atypical movement parameters. Some observed differences in lumbo-pelvic kinematic parameters for those with and without LBP appear both clinically relevant and biologically plausible.

## Additional files

Additional file 1: Appendix 1. Description and image of the lumbar ‘classifier’.questionnaire. (DOCX 154 kb)

Additional file 2: Appendix 2. Description and details of measured lumbo-pelvic kinematics. (DOCX 14 kb)

## Abbreviations

FRR: Flexion relaxation response
LBP: Low Back Pain
NoLBP: participants without low back pain
RMDQ: Roland Morris Disability Questionnaire
ROM: Range of movement
