# Supplementary material for: Does movement matter in people with back pain? Investigating ‘atypical’ lumbo-pelvic kinematics in people with and without back pain using wireless movement sensors
Source: BMC Musculoskelet Disord. 2019 Jan 18;20:28. doi: 10.1186/s12891-018-2387-x (PMC6339318; doi:10.1186/s12891-018-2387-x)

## Additional file 1: Appendix 1

### Lumbar ‘classifier’ questionnaire.

Only question 1 was used to provide a score for pain on bending, and question 11 and 12 for pain on sitting.


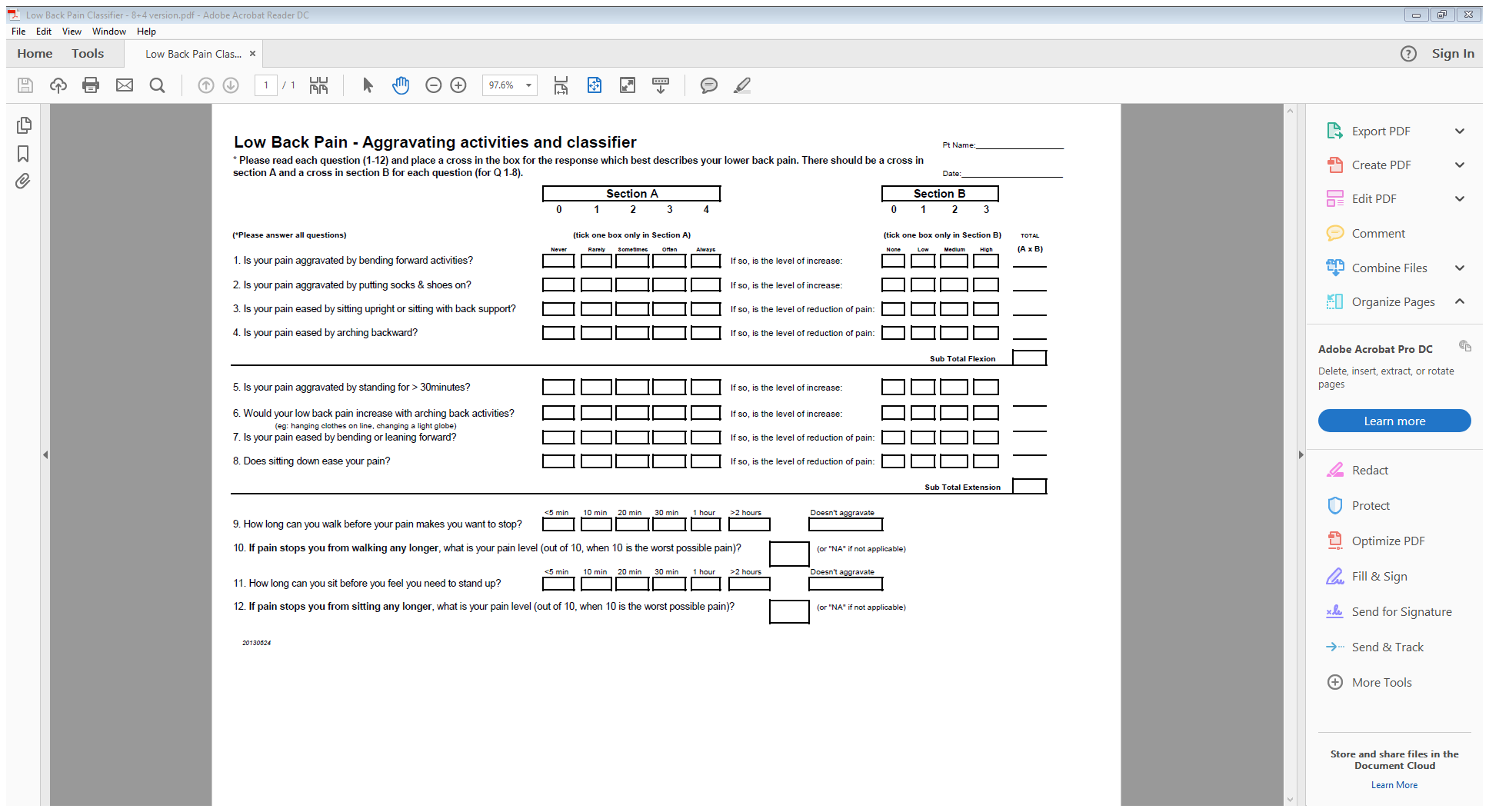

Supplement: Supplementary file 1 — Appendix 1. Description and image of the lumbar ‘classifier’.questionnaire. (DOCX 154 kb) [file 12891_2018_2387_MOESM1_ESM.docx]
