# Supplementary material for: Does movement matter in people with back pain? Investigating ‘atypical’ lumbo-pelvic kinematics in people with and without back pain using wireless movement sensors
Source: BMC Musculoskelet Disord. 2019 Jan 18;20:28. doi: 10.1186/s12891-018-2387-x (PMC6339318; doi:10.1186/s12891-018-2387-x)
Supplement: Supplementary file 2 — Appendix 2. Description and details of measured lumbo-pelvic kinematics. (DOCX 14 kb) [file 12891_2018_2387_MOESM2_ESM.docx]

### Description and details of measured lumbo-pelvic kinematics

Each participant was fitted with the wireless motion and surface EMG sensors as described in the method section. The initial calibration of lordosis was calculated by the absolute angular inclination against a vertical line determined by gravity. The sensors were then calibrated to a ‘zero’ position so that all movements started from a zeroed position. Integrity of motion and EMG was tested by performing a single practice movement of flexion while the clinician observed the output on the computer screen.

| **Description** | **Movement characteristics recorded** | **Details and instructions** |
| --- | --- | --- |
| Flexion ROM, three repetitions | Angular inclination of trunk (at T12) in degrees  Angular inclination of pelvis (at S2) in degrees  Lumbar spine ROM (T12 angle minus S2 angle)  Lumbar contribution to flexion (%)*  Flexion relaxation response (calculated as a ratio of sEMG activity – see Figure 2)  Delay (lag) of pelvic versus lumbar timing at the start  Delay (lag) of pelvic versus lumbar at 20o of movement  Duration of bending motion | “Move from neutral standing to full flexion (towards your toes), at your own speed, hold for 3 seconds, then return to neutral”  The time count of three seconds was provided. Each person had a test run of a single movement to ensure accurate calibration i.e. the sensors returned to a zeroed position. Data was then captured for 3 repetitions of flexion. |
| Usual sitting position (15 seconds with 5 seconds of captured data) | Trunk angle and pelvic angle  Lumbar ROM (Angular inclination at T12 minus angular inclination at S2)  ‘Usual” sitting position calculated as a percentage where full posterior tilt = 100% and full upright sitting = 0% | No instructions, people were asked to sit on a 55cm or 65 cm ball (hips slightly higher than knees) while the assessor “worked on the computer” for 10 seconds to allow for a stable position to be established with minimal change, then 5 seconds of data captured). |
| Upright sitting position | Trunk angle and pelvic angle  Lumbar ROM (Angular inclination at T12 minus angular inclination at S2) | The participant was asked to “sit upright as much as possible” for 10 seconds, then 5 seconds of data captured). |
| Slumped sitting position | Trunk angle and pelvic angle  Lumbar ROM (Angular inclination at T12 minus angular inclination at S2) | The participant was asked to “sit in their tired, slumped, relaxed position” for 10 seconds, then 5 seconds of data captured). |
